# Development of a cellular model to study CCR8 signaling in tumor-infiltrating regulatory T cells

**DOI:** 10.1007/s00262-023-03607-z

**Published:** 2024-01-17

**Authors:** Libao Liu, Laurie Rangan, Nathan Vanalken, Qianqian Kong, Susan Schlenner, Steven De Jonghe, Dominique Schols, Tom Van Loy

**Affiliations:** 1 Laboratory of Virology and Chemotherapy, Department of Microbiology, Immunology and Transplantation, Rega Institute for Medical Research, KU Leuven, B-3000 Leuven, Belgium; 2 Laboratory of Adaptive Immunology, Department of Microbiology, Immunology and Transplantation, KU Leuven, B-3000 Leuven, Belgium

**Keywords:** Tumor-infiltrating regulatory T cells, Cellular model, TITR mimics, CCR8, Small molecules, Chemotaxis

## Abstract

The human CC chemokine receptor 8 (CCR8) is specifically expressed on tumor-infiltrating regulatory T cells (TITRs) and is a promising drug target for cancer immunotherapy. However, the role of CCR8 signaling in TITR biology and the effectiveness of CCR8 small molecule antagonists as TITR-targeting immunotherapy remain subjects of ongoing debate. In this work, we generated a novel cellular model of TITRs by culturing peripheral blood mononuclear cell-derived regulatory T cells in medium containing tumor cell-conditioned medium, CD3/CD28 activator, interleukin-2 and 1α,25-dihydroxyvitamin D3. This cellular model (named TITR mimics) highly and stably expressed a series of TITR signature molecules, including CCR8, FOXP3, CD30, CD39, CD134, CD137, TIGIT and Tim-3. Moreover, TITR mimics displayed robust in vitro immunosuppressive activity. To unravel the functional role of CCR8 in TITR mimics, a chemotaxis assay was performed showing strong and CCR8-specific migration toward CCL1, the natural chemokine agonist of CCR8. However, either stimulation (with CCL1) or blocking (with the small molecule antagonist NS-15) of CCR8 signaling did not affect the immunosuppressive activity, proliferation and survival of TITR mimics. Collectively, our work provides a method for the generation of TITR mimics in vitro, which can be used to study TITR biology and to evaluate drug candidates targeting TITRs. Furthermore, our findings suggest that CCR8 signaling primarily regulates migration of these cells.

**Supplementary Information:**

The online version contains supplementary material available at 10.1007/s00262-023-03607-z.

## Introduction

It was unveiled in 2016 that CCR8 is specifically expressed on tumor-infiltrating regulatory T cells (TITRs) across a range of primary and metastatic human cancer types, including breast, lung, colon carcinoma, and melanoma. Moreover, the accumulation of CCR8^+^ TITRs was shown to correlate with poor survival rates among patients [1, 2]. Consequently, the development of therapeutic antibodies targeting CCR8 has been pursued as a potential approach for cancer treatment. Particularly, CCR8 antibodies capable of inducing antibody-dependent cellular cytotoxicity (ADCC) have demonstrated their ability to selectively eliminate TITRs within tumor sites, resulting in reduced tumor growth without causing side effects [3–6]. Additionally, as a standalone therapy, these antibodies have been found to be effective in slowing down the growth of anti-PD1 resistant tumors, and when co-administrated, they can effectively revive the efficacy of anti-PD1 treatment [4, 6]. These findings highlight the promising potential of CCR8 as a target on TITRs for cancer treatment, even in patients with resistance to immune checkpoint inhibitors (ICIs).

While CCR8 has been recognized as a specific marker of TITRs, the role of CCR8 signaling in TITRs remains a subject of debate. Studies utilizing CCR8 knockout mice revealed no alteration in the recruitment, activation, and immunosuppressive capacity of TITRs, and no impact on tumor growth [3, 7]. Likewise, CCR8-blocking antibodies lacking ADCC effect showed no efficacy in suppressing tumor growth [3–6]. However, a recent study highlighted the inhibitory effect of IPG7236, a selective small molecule CCR8 antagonist, on tumor growth [8]. Furthermore, in vitro studies on this matter have also produced conflicting results. While some studies suggest that CCR8 signaling does not impact TITR suppressor function [1, 3], others have reached the opposite conclusion [9].

The high level of CCR8 expression on freshly isolated TITRs suggests that CCR8 expression on TITRs is persistent and stable in the tumor microenvironment. However, the stability of CCR8 expression in ex vivo cultured TITRs has not been clearly established in previous studies. Moreover, there is a lack of characterization of other TITRs signature molecules in these ex vivo TITRs [1, 9]. Therefore, up to now, it remains unclear how well ex vivo TITRs represent the in vivo TITRs, thereby raising concerns about the efficacy of previous research on the role of CCR8 in TITRs. In addition, due to the scarcity of patient tumor samples, human TITRs may not be easily accessible, especially to academic researchers. Hence, there is a clear need to establish a well-characterized, easily available, and physiologically relevant cellular model for studying TITRs.

In this study, we developed a method to generate TITR mimics from peripheral blood mononuclear cell (PBMC)-derived Tregs, employing CD3/CD28 activator, interleukin 2 (IL-2), vitamin D3 (VitD3) and tumor cell-conditioned medium (TCM). The obtained TITR mimics exhibited sustained high expression of CCR8. Moreover, these TITR mimics also showed significantly upregulated expression of several selected TITR signature molecules and displayed robust immunosuppressive activity. Whereas these TITR mimics specifically responded to CCL1, the natural chemokine agonist of CCR8, in a cellular migration assay, CCR8 activation or blockade did not affect their immunosuppressive capability, proliferation and survival. These results suggest that CCR8 signaling mainly (co-)regulates the migration and positioning of these cells, but is likely dispensable for their proliferation, survival and immunosuppressive phenotype, at least when studied in vitro without the presence of potential accessory cells.

## Materials and methods

### Reagents, compounds and antibodies

ImmunoCult™-XF T cell expansion medium (XF medium; #10,981), ImmunoCult™ human CD3/CD28 T cell activator (CD3/CD28 activator; #10,971) and rapamycin (#73,362) were purchased from STEMCELL Technologies. 1α,25-Dihydroxyvitamin D3 (VitD3; #D1530) was obtained from Sigma-Aldrich. Recombinant human interleukin-2 (IL-2; #202-IL) and chemokine CCL1 (#272-I) were obtained from R&D systems. The CCR8-targeting small molecule NS-15 [10] was kindly provided by professor W. Dehaen (Department of Chemistry, KU Leuven). CCL1^AF647^ (#CAF-07) was obtained from Almac. Antibodies targeting CD30 (#333,909), CD39 (#328,209), CD80 (#375,403), CD120b (#358,405), CTLA-4 (#369,611), TIGIT (#372,705), Tim-3 (#345,011), GITR (#311,609), ICOS (#313,509), CCR8 (#360,604), granzyme B (#515,406) and corresponding isotype antibodies were purchased from Biolegend. Antibodies targeting CD134 (#555,838), CD137 (#555,956), RORgt (#563,081) and corresponding isotype antibodies were obtained from BD Biosciences. FOXP3 (#2,200,560), IL-10 (#3,134,055), IL-17 (#3,161,615) were obtained from Sony.

### Tregs and responder T cells (Tresps)

Human peripheral blood mononuclear cells (PBMCs) were obtained from the Red Cross (Biobank Rode Kruis—Vlaanderen). Both Tregs and Tresps were isolated from fresh PBMCs using the EasySep™ human CD4^+^CD127^low^CD25^+^ regulatory T cell isolation kit (STEMCELL Technologies, #18,063). The freshly isolated Tregs were then expanded using a previously established protocol [11], but with minor adaptations. Briefly, on day 0, isolated Tregs were seeded in a 12-well plate at ~ 1.5 × 10^5^ cells per mL per well in XF medium supplemented with 25 µL/mL CD3/CD28 activator and 100 ng/mL rapamycin. On day 2, the culture medium volume was tripled by adding 2 mL XF medium. Rapamycin (100 ng/mL) and 300 IU/mL IL-2 were added on day 2, 5, and 7 (assuming consumption). The expanded Tregs were used from day 9 on. Allogeneic CD4^+^CD25^−^ responder T cells (Tresps) were isolated on day 7 and then rested in XF medium for 2 days until use on day 9. Both Tregs and Tresps were characterized with CD4/CD25/CD127 staining (Fig. S1).

### Tumor cell-conditioned medium

2 × 10^6^ MDA-MB-231 cells (human breast cancer cell line, ATCC) or A549 cells (human lung cancer cell line, ATCC) per T75 flask were grown for 3–4 days in 20 mL of Dulbecco’s Modified Eagle Medium (DMEM) containing 10% fetal bovine serum (FBS). The tumor cell-conditioned medium (TCM) was then harvested and stored at − 20 °C after removal of cell debris by centrifugation at 400 × g for 5 min at room temperature (RT). When used, TCM was dissolved at RT and then mixed with XF medium at a ratio of 1:1 immediately before use. For convenience, TCM derived from MDA-MB-231 and A549 cells is designated as TCM231 and TCM549, respectively. When combined with XF medium, they are identified as TCM231-XF and TCM549-XF medium, respectively.

### Method optimization for generating TITR mimics

To optimize the generation of TITR mimics, eight distinct cell culture methods were examined side-by-side. This includes the use of XF medium and TCM231-XF medium supplemented with various combinations of CD3/CD28 activator, IL-2 and VitD3. When applied, the concentrations used for CD3/CD28 activator, IL-2, and VitD3 were 12.5 µL/mL, 300 IU/mL, and 10 nM, respectively. Tregs expanded for 9 days were washed once with XF medium and then cultured in a flat-bottom 96-well plate (Corning, #353,072) at 7.5 × 10^4^ cells per well in 200 µL volume for 24 h, or in T25 flasks at 5 × 10^5^ cells per flask in 5 mL volume for 72 h using the different culture media mentioned above. The obtained Tregs were then characterized by flow cytometry for the expression of multiple TITR-related molecules, including CCR8, CD30, CD39, CD134, CD137, TIGIT and Tim-3, with quantification of the percentage and median fluorescence intensity (MFI) of cells expressing these molecules using FlowJo software version 10.8.1.

### CCL1^AF647^ binding assay

Expanded Tregs were grown in TCM231-XF medium with 12.5 µL/mL CD3/CD28 activator, 300 IU/mL IL-2 and 10 nM VitD3 for 24 h. Cells were then treated with CCR8 ligands (NS-15 at 1 µM, and CCL1 at 50/100/200 ng/mL) for either 30 min, 24 h or 48 h (in this case ligands were added twice, at 24 h and 48 h), respectively, followed by staining with 3 nM CCL1^AF647^. Cells were thus stained at 24.5 h, 48 h or 72 h, respectively. For CCL1^AF647^ staining, without washing, the cells were incubated with 3 nM CCL1^AF647^ for 30 min at RT in the dark. The cells were then washed twice and resuspended in Hanks’ Balanced Salt Solution (HBSS)-based buffer (HBSS + 20 mM HEPES + 0.5% FBS, pH 7.4) for flow cytometry analysis. The percentage of CCL1^AF647^-bound cells was quantified using FlowJo software version 10.8.1.

### Transwell migration assay

A Transwell migration assay was performed using 6.5 mm Transwell® inserts with 5.0 µm pore polycarbonate membranes (Corning, #3421). After 9 days of expansion, Tregs were washed once with XF medium and then re-stimulated for 24 h with 12.5 µL/mL CD3/CD28 activator, 300 IU/mL IL-2, 10 nM VitD3 in TCM231-XF medium. The cells were then washed twice and resuspended in HBSS-based assay buffer to assess the expression of CCR8 and CCR4, as well as to conduct the Transwell migration assay. To set up the Transwell plate, 2.5 × 10^5^ cells (100 µL) were added to the upper wells, while the lower wells received 600 µL of HBSS-assay buffer containing CCR8 and/or CCR4 ligands. The ligands being investigated include CCR8 agonist CCL1 at 5.88 nM (equal to 50 ng/mL), CCR8 antagonist NS-15 at 1 µM and CCR4 agonist CCL22 at 5.88 nM. All the upper and lower wells have the same concentration of vehicles, including DPBS for chemokines and DMSO for small molecule antagonist. The plate was then transferred to 37 °C and 5% CO_2_ for 2 h. Following incubation, the number of cells in 400 µL of suspension from the lower wells was quantified by flow cytometry (FACS CantoII) and used to calculate the migration percentage.

### In vitro Treg suppression assay

Tresps were labeled with 2 µM of CellTrace Violet (Invitrogen, #C34571) following the manufacturer’s protocol. Afterward, the labeled Tresps were mixed with expanded Tregs and cultured for 4 days using flat-bottom 96-well plates in TCM231-XF medium supplemented with 12.5 µL/mL CD3/CD28 activator, 300 IU/mL IL-2, 10 nM VitD3, with a fixed total number of 1 × 10^5^ cells in 200 µL per well. To determine the dose-dependent immunosuppressive activity of Tregs, expanded Tregs were mixed with labeled Tresps at ratios ranging from 1:4 to 1:64, with labeled Tresps in the absence of Tregs used as proliferating control. To investigate the effect of CCR8 ligands, labeled Tresps were cultured either alone or in the presence of expanded Tregs (Treg:Tresps = 1:16) and treated with CCR8 ligands (NS-15 at 1 µM, and CCL1 at 50/100/200 ng/mL) at 24, 48, and 72 h. The untreated cell mixtures were used as control. After approximately 96 h, the cells were washed twice and resuspended in HBSS-based buffer for measurement of CellTrace Violet signal using flow cytometry to track cell proliferation, which was quantified using the division index (DI) generated by FlowJo’s proliferation modeling tool.

### Treg proliferation assay

Expanded Tregs were labeled with 2 µM of CellTrace Violet according to the manufacturer’s instructions and then cultured at 1 × 10^5^ cells per 200 µL per well in flat-bottom 96-well plates for 4 days using TCM231-XF medium supplemented with 12.5 µL/mL CD3/CD28 activator, 300 IU/mL IL-2, 10 nM VitD3. CCR8 ligands (NS-15 at 1 µM, and CCL1 at 50/100/200 ng/mL) were added to the cell culture at 24, 48 and 72 h. After 96 h, the cells were washed twice and resuspended in HBSS-based buffer for flow cytometry analysis. The proliferation of labeled Tresps was quantified as described above.

### Treg survival assay

After 9 days of expansion, Tregs were grown in TCM231-XF medium supplemented with 12.5 µL/mL CD3/CD28 activator, 300 IU/mL IL-2, 10 nM VitD3 for another 3 days. The obtained cells were labeled with 1 µM of CellTrace Violet for cell tracking and then exposed to CCR8 ligands (NS-15 at 1 µM, and CCL1 at 50/100/200 ng/mL) in XF medium for 24 h, either with or without the presence of 2 µg/mL of CD95 antibodies (Biolegend, #305,705). Afterward, the cells were stained with APC Annexin V (Biolegend, #640,920) following the manufacturer’s protocol and analyzed by flow cytometry. The survival rate of the CellTrace Violet-labeled cells was presented.

### Statistics

All graphs show the mean and standard deviation (mean ± SD) of *n* biological replicates. Statistical significance was determined using GraphPad Prism software (version 9.5.1). For pairwise comparisons, paired *t* test was performed. One-way analysis of variance (ANOVA) followed by a Tukey test was performed to compare each condition with every other condition. For statistically significant differences, the p value is indicated in graphs as the following: *p < 0.05, **p < 0.01, ***p < 0.001, ****p < 0.0001.

## Results

### The simultaneous use of CD3/CD28 activator, IL-2, VitD3 and TCM231 induces high and stable expression of CCR8 in Tregs

Previous studies have established that CD3/CD28 activation is crucial for the expression of CCR8 [1, 3]. FOXP3 has also been implicated in CCR8 regulation [12]. As IL-2 has the ability to enhance FOXP3 expression in Tregs [13], it may also facilitate CCR8 expression in an indirect manner. Moreover, VitD3 has been shown to induce CCR8 expression in naïve T cells and Teff cells [14, 15]. Given the high expression of the VitD3 receptor (VDR) in TITRs [1, 16], VitD3 may also contribute to the upregulation of CCR8 in Tregs. Finally, co-culture with breast tumor slides was found to substantially upregulate CCR8 mRNA in CD3/CD28-activated Tregs [1]. Since TCM has soluble factors produced by cancer cell lines, we hypothesized that TCM may be used as an alternative to enhance CCR8 expression in Tregs.

To optimize the upregulation of CCR8 expression, PBMC-derived expanded Tregs were cultured in XF medium or TCM-231-XF medium with various combinations of CD3/CD28 activator, IL-2 and VitD3. The expression of CCR8 was analyzed at 24 and 72 h to investigate the time required for CCR8 induction and the duration of its expression. CD3/CD28 activation by itself slightly enhanced CCR8 expression in Tregs cultured in both media at 24 h, even though this effect was not statistically significant. Administration of IL-2 further enhanced the number of CCR8^+^ cells significantly. Statistical comparison between the conditions XF-CD3/CD28 activator with or without IL-2 revealed a significant increase in the number of CCR8^+^ cells at both 24 h (*p* = 0.0198) and 72 h (*p* = 0.0006). When cultured in TCM231-CD3/CD28 activator, a similar effect of IL-2 on the number of CCR8^+^ cells was observed at 72 h (*p* < 0.0001). When cultured in XF medium the application of VitD3 led to a significant additional increase in the number of CCR8 expressing cells (when compared to XF-CD3/CD28 activator-IL-2) at 72 h (*p* = 0.0016), but not at 24 h (*p* = 0.5010). Although the effect of VitD3 was less pronounced in TCM231-XF medium and was not statistically significant at 72 h (*p* = 0.4953) a slight increase in the number of CCR8^+^ cells was still observed in all three independent experiments. Furthermore, when supplemented with the same combination of CD3/CD28 activator, IL-2 and VitD3, TCM-XF medium consistently yielded a greater number of CCR8^+^ cells in comparison to XF medium at 72 h in all three independent experiments with significant differences observed between conditions containing CD3/CD28 activator (*p* = 0.0088) and CD3/CD28 activator plus IL-2 (*p* = 0.0005).

**Fig. 1:**
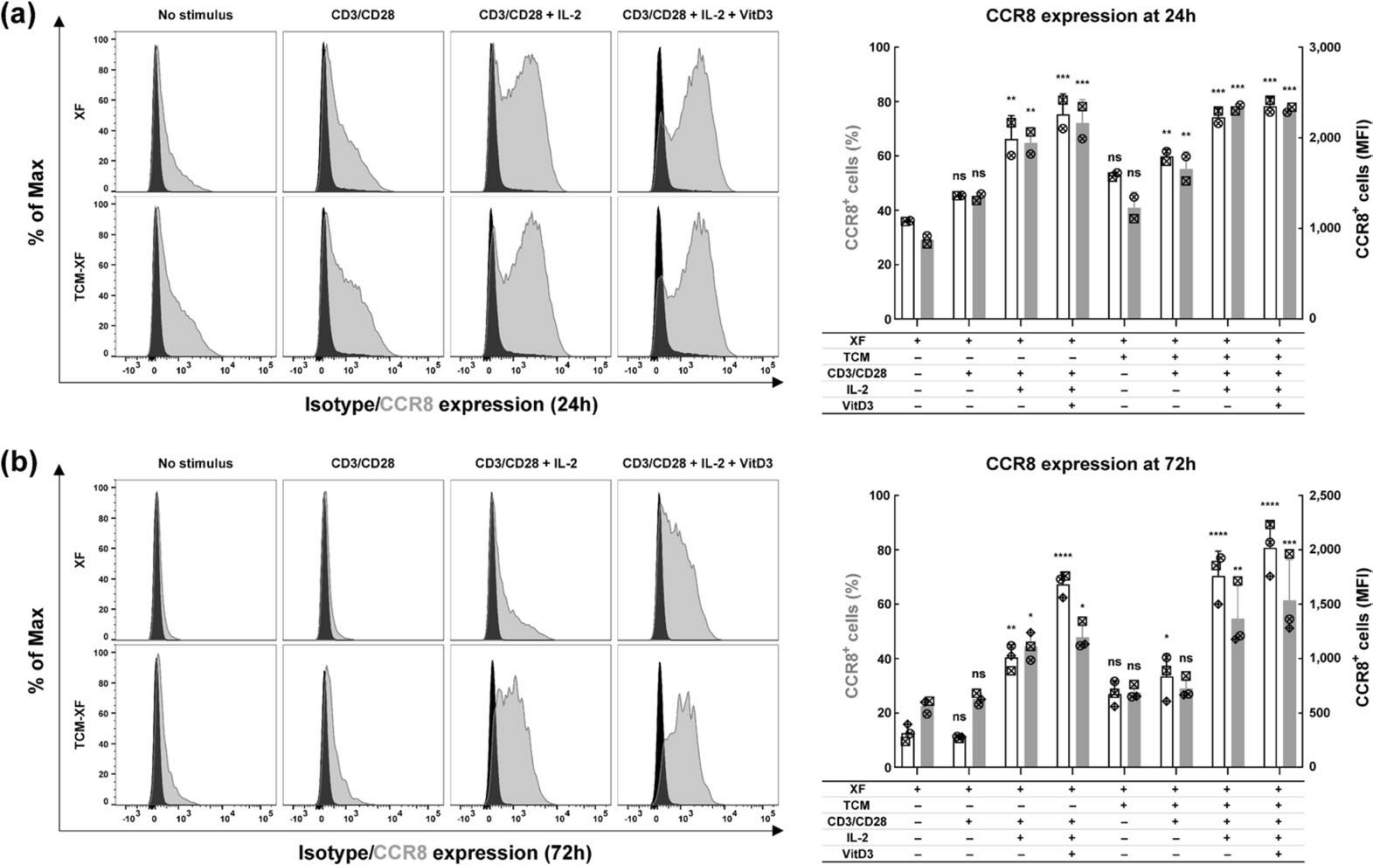
CCR8 is upregulated by simultaneous use of CD3/CD28 activator, IL-2, VitD3 and TCM231. **a** CCR8 expression in Tregs after 24 h of culture. After expansion for 9 days, Tregs were cultured at 7.5 × 10^4^ cells per well in a flat-bottom 96-well plate in 200 µL of XF medium or TCM231-XF medium containing different combinations of CD3/CD28 activator, IL-2 and VitD3 for 24 h. Left histograms show the representative donor staining with isotype (black) and CCR8 antibody (gray). Right bars show the percentage of CCR8^+^ Tregs (white) and MFI (gray) on CCR8^+^ Tregs (*n* = 2). **b** CCR8 expression in Tregs after 72 h of culture. After expansion for 9 days, Tregs were cultured at 5 × 10^5^ cells per T25 flask in 5 mL of XF medium or TCM231-XF medium containing different combinations of CD3/CD28 activator, IL-2 and VitD3 for 72 h. Left histograms show the representative donor staining with isotype (black) and CCR8 antibody (gray). Right bars show the percentage (white) and MFI (gray) of CCR8^+^ Tregs (*n* = 3). For bar graphs, a single independent experiment is represented by data points of the same shape. Data shown as mean ± SD. One-way ANOVA followed by Tukey test was performed to compare each condition with every other condition. For clarity, only the difference between XF medium and every other condition is shown (* *p* ≤ 0.05, ** *p* ≤ 0.01, *** *p* ≤ 0.001 and **** *p* ≤ 0.0001). ANOVA, analysis of variance; MFI, median fluorescence intensity. Histograms were generated as SVG files using FlowJo software. Bar graphs were created using GraphPad Prism. The alignment of the histograms and bar graphs was performed using the open-source Inkscape software

Taken together, these findings revealed that all the tested factors (CD3/CD28 activator, IL-2, VitD3 and TCM231) can contribute to the upregulation of CCR8 in Tregs, with TCM and IL-2 being most important to obtain stable CCR8 expression. The less pronounced impact of VitD3 in TCM231-XF medium might suggest that some levels of VitD3 are already present in TCM231. Of note, the simultaneous use of the four factors not only increased the number of CCR8-expressing cells (reaching approximately 80%), but also upregulated CCR8 abundance on these cells (resulting in up to threefold increase in MFI) (Fig. 1).

#### CD3/CD28 activator, IL-2, VitD3 and TCM231 also upregulate the expression of other TITR signature molecules

**Fig. 2:**
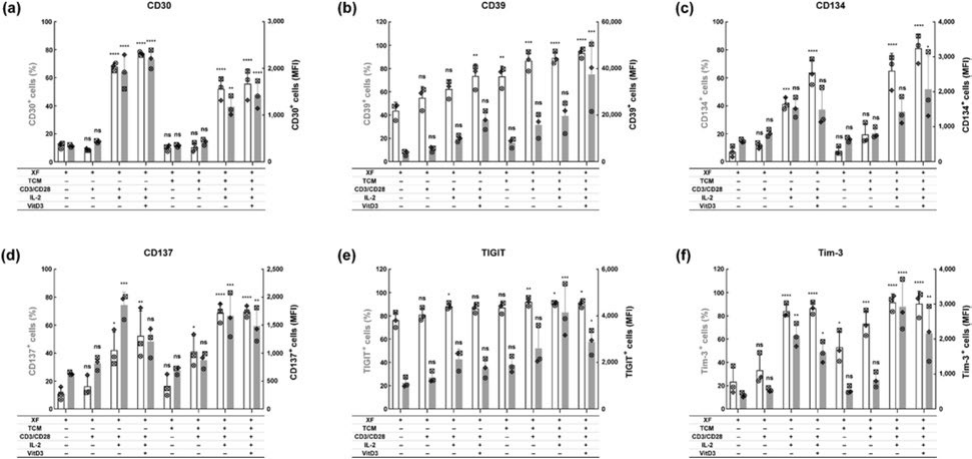
The simultaneous application of CD3/CD28 activator, IL-2, VitD3 and TCM231 significantly increases the expression of selected TITR signature molecules in Tregs. After expansion for 9 days, Tregs were cultured at 5 × 10^5^ cells per T25 flask in 5 mL of XF medium or TCM231-XF medium containing different combinations of CD3/CD28 activator, IL-2 and VitD3 for 72 h. Bars show the percentage (white) and MFI (gray) of cells expressing CD30 (**a**), CD39 (**b**), CD134 (**c**), CD137 (**d**), TIGIT (**e**) and Tim-3 (**f**). A single independent experiment is represented by data points of the same shape (*n* = 3). Data are mean ± SD. One-way ANOVA followed by Tukey test was performed to compare each condition with every other condition. The differences between XF medium and every other condition are shown (* *p* ≤ 0.05, ** *p* ≤ 0.01, *** *p* ≤ 0.001 and **** *p* ≤ 0.0001). ANOVA, analysis of variance; MFI, median fluorescence intensity. Histograms were generated as SVG files using FlowJo software. Bar graphs were created using GraphPad Prism. The alignment of the histograms and bar graphs was performed using the open-source Inkscape software

To further characterize the Tregs, we assessed the expression of several molecules previously reported to be upregulated in TITRs, including CD30, CD39, Tim-3, TIGIT, CD134, and CD137, after 72 h of treatment [1, 17]. Expression of these molecules was limited in untreated Tregs when cultured in XF medium. Only about 20% of cells expressed CD30, CD134, CD137, Tim-3 and around 40% showed CD39 expression. Although TIGIT expression was detected in approximately 80% of untreated Tregs, the level of expression on these cells was relatively low (Fig. 2 and Fig. S2). Treatment with CD3/CD28 activator slightly increased the expression of all these tested molecules, except for CD30. Co-administration of CD3/CD28 activator and IL-2 significantly boosted the expression of all molecules. VitD3 further upregulated CD30, CD39 and CD134, but it slightly decreased the expression of CD137, TIGIT, and Tim-3. Compared to Tregs cultured in XF medium, Tregs in TCM231-XF medium receiving the same treatments displayed more pronounced expression of all these molecules, with exception of CD30. Of note, even though VitD3 and TCM231 were not always favorable, the combination of CD3/CD28 activator, IL-2, VitD3 and TCM231 still greatly augmented the expression of all the TITRs signature molecules under investigation (Fig. 2 and Fig. S2).

To further authenticate the Tregs, we cultured the cells for 4 days in TCM231-XF medium containing CD3/CD28 activator, IL-2 and VitD3. During the last 4 h of the culture, 50 ng/mL Phorbol 12,13-dibutyrate (Tocris, #4153) and 1 µg/mL Ionomycin (Tocris, #1704) were introduced in the presence of 1 µg/mL Brefeldin-A. The cells were then stained for FOXP3, IL-10 and IL-17. The results revealed that the vast majority of Tregs expressed a high level of FOXP3, while small (sub-)populations also expressed IL-10 and IL-17 (Fig. S3).

### TITR mimics display robust immunosuppressive activity in vitro

**Fig. 3:**
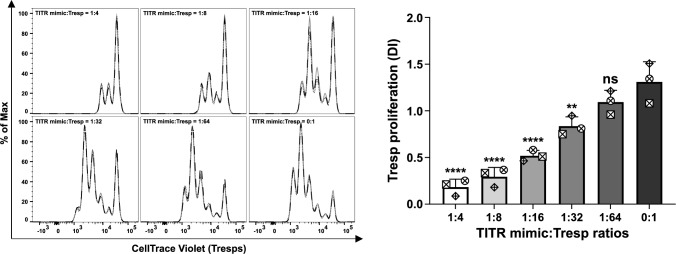
TITR mimics display dose-dependent immunosuppressive activity in in vitro suppression assay. Allogeneic Tresps were labeled with 2 µM CellTrace Violet and then co-cultured with Tregs that underwent 9 days of expansion at different ratios, with a fixed total number of 1 × 10^5^ per well of 96-well plate, in TCM231-XF medium containing 12.5 μg/mL CD3/CD28 activator, 300 IU/mL IL-2 and 10 nM VitD3 for 4 days. Left histograms show the proliferation of Tresps from one representative experiment with three technical replicates. Right bars show the division index (DI) of Tresps calculated from three biological replicates (each performed in triplicate) by FlowJo proliferation modeling. For bar graphs, a single independent experiment is represented by data points of the same shape (*n* = 3). Data shown as mean ± SD. * *p* ≤ 0.05, ** *p* ≤ 0.01, *** *p* ≤ 0.001 and **** *p* ≤ 0.0001 by one-way ANOVA where each ratio was compared with the TITR mimic:Tresp ratio of 0:1. ANOVA, analysis of variance. Histograms were generated as SVG files using FlowJo software. Bar graphs were created using GraphPad Prism. The alignment of the histograms and bar graphs was performed using the open-source Inkscape software

Next, we investigated the immunosuppressive capacity of Tregs cultured in TCM231-XF medium supplemented with CD3/CD28 activator, IL-2 and VitD3. For this purpose, we cultured CellTrace Violet-labeled allogeneic CD4^+^CD25^−^ T cells as responder cells (Tresps) for 4 days in the absence or presence of Tregs. As shown in Fig. 3, robust proliferation of Tresps was observed in the absence of Tregs. When co-cultured, Tregs suppressed the proliferation of Tresps in a dose-dependent manner. Of note, even at physiologically relevant Treg:Tresp ratios of 1:16 or lower, proliferation of Tresps was still efficiently suppressed.

A comparison was also carried out to assess the suppressive capacity of Tregs cultured in TCM231-XF medium compared to those cultured in XF-medium. TCM231-XF medium by itself has the potential to lower Tresp cell proliferation when compared with XF medium (Fig. S4a and S4b). Notably, the analysis of the inhibition percentage revealed that Tregs in TCM-XF medium possessed stronger immunosuppressive capacities compared to those in XF medium (Fig. S4c). The strong immunosuppressive capabilities of the Tregs (when cultured in TCM231-XF medium containing CD3/CD28 activator, IL-2 and VitD3), along with their phenotypic similarity to TITRs, led us to designate them as “TITR mimics”.

### TITR mimics are able to migrate in a CCR8-dependent manner

**Fig. 4:**
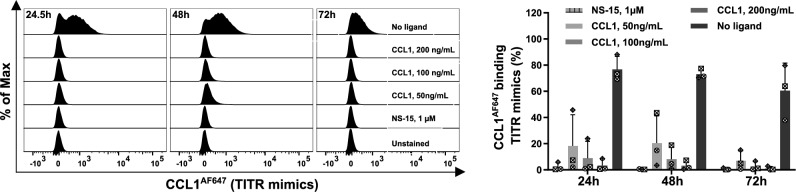
CCR8 ligands effectively block CCL1^AF647^ binding on TITR mimics. TITR mimics were cultured at 5 × 10^4^ cells per well in a 96-well plate in TCM231-XF medium containing 12.5 μg/mL CD3/CD28 activator, 300 IU/mL IL-2 and 10 nM VitD3. CCR8 ligands at the indicated concentrations were added at 24 h, and then, cells were stained with 3 nM CCL1^AF647^ 30 min later (24.5 h) and 24 h later (48 h), respectively. Other cells were treated with CCR8 ligands twice (at 24 and 48 h, respectively) and then stained with 3 nM CCL1^AF647^ at 72 h. Left histograms show representative staining with CCL1^AF647^. Right bar graphs show the percentage of CCL1^AF647^ binding cells. For bar graphs, a single independent experiment is represented by data points of the same shape (*n* = 3). Data shown as mean ± SD. Histograms were generated as SVG files using FlowJo software. Bar graphs were created using GraphPad Prism. The alignment of the histograms and bar graphs was performed using the open-source Inkscape software

TITR mimics were then used as a cellular model to study the involvement of CCR8 signaling in the immunosuppressive function of TITRs. To this end, the cognate ligand CCL1 was used to activate CCR8 signaling. In addition, considering that activated T cells are known to produce CCL1 [18], the effect of blockade of CCR8 activation by autocrine and paracrine CCL1 was also examined using the small molecule antagonist NS-15 [19]. The interaction of CCL1 and NS-15 with CCR8 expressed on TITR mimics was first confirmed through a binding assay involving CCL1^AF647^. As shown in Fig. 4, CCL1 dose-dependently inhibits CCL1^AF647^ binding. Furthermore, NS-15 at 1 µM almost fully blocked the binding of CCL1^AF647^.

**Fig. 5:**
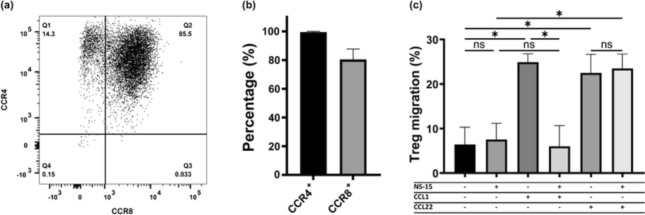
CCR8 on TITR mimics is functional in a Transwell migration assay. **a** Representative flow cytometry dot plot showing CCR8 and CCR4 expression on TITRs mimics, stained by BV421-CCR8 (BD, #566,379) and APC-CCR4 (Biolegend, #359,408). **b** Percentage of TITR mimics expressing CCR8 and CCR4 (*n* = 2). **c** Migration of TITR mimics toward CCL1 and CCL22 in the presence or absence of NS-15 (*n* = 2). One-way ANOVA followed by Tukey test was performed to compare each condition with every other condition. The differences between selected pairs were shown, * *p* ≤ 0.05

Chemokine receptors are primarily known for their role in regulating the homing and migration of immune cells. CCR8 being highly expressed on TITR mimics therefore naturally suggests a role in their migration upon interaction with the natural CCR8 agonist CCL1. Previously, the chemokine receptor CCR4 was also reported to be highly expressed by TITRs [1, 20–22]. We therefore first assessed expression of both CCR4 and CCR8 in the TITR mimics, which demonstrated that the vast majority of these cells co-express both receptors (Fig. 5a, b). In a Transwell migration assay, both CCL1 (CCR8 agonist) and CCL22 (CCR4 agonist) induced strong TITR mimic cell migration. To further demonstrate that the CCL1-induced migration was uniquely mediated by CCR8, the small molecule CCR8 antagonist NS-15 was applied. Whereas NS-15 was unable to induce cell migration by itself, it fully inhibited the CCL1-induced migration response (Fig. 5c). In contrast, NS-15 had no effect on CCL22-induced migration (Fig. 5c).

### CCR8 signaling is dispensable for the immunosuppressive activity of TITR mimics

**Fig. 6:**
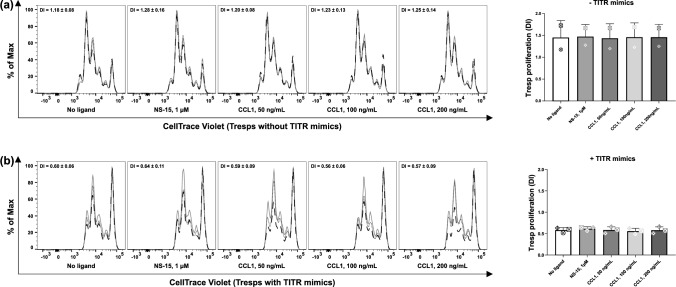
CCR8 is redundant for the immunosuppressive activity of TITR mimics. **a** The effects of CCR8 ligands on Tresp proliferation in the absence of TITR mimics. **b** The effects of CCR8 ligands on Tresp proliferation in the presence of TITR mimics. Tresps were labeled with 2 µM CellTrace Violet and then culture alone or in the presence of TITR mimics at a ratio of one Treg for sixteen Tresps, with a total number of 1 × 10^5^ cells per well in a 96-well plate, in TCM231-XF medium containing 12.5 μg/mL CD3/CD28 activator, 300 IU/mL IL-2 and 10 nM VitD3 for 4 days. Cells received CCR8 ligands at indicated concentrations at 24 h, 48 h and 72 h and then analyzed at 96 h. Left histograms show the Tresp proliferation in single representative experiments with three technical replicates. Right bars show the division index (DI) calculated from two or three biological replicates (each performed in triplicate) of proliferating Tresps. A single independent experiment is represented by data points of the same shape. Data shown as mean ± SD. Histograms were generated as SVG files using FlowJo software. Bar graphs were created using GraphPad Prism. The alignment of the histograms and bar graphs was performed using the open-source Inkscape software

To investigate the role of CCR8 signaling in the immunosuppressive function of TITR mimics, CellTrace Violet-labeled allogeneic Tresps were cultured with (Treg:Tresp = 1:16) or without TITR mimics in TCM231-XF medium containing CD3/CD28 activator, IL-2 and VitD3 for 4 days. CCL1 and NS-15 were added to the culture at 24 h, 48 h and 72 h. The results showed that the proliferation of Tresps remained unaffected by either CCR8 activation via CCL1 or blockade by NS-15, regardless of the presence or absence of TITR mimics (Fig. 6a, b). Hence, at least in our in vitro system, it can be inferred that CCR8 signaling is not essential for the immunosuppressive capability of TITR mimics.

### CCR8 signaling is not necessary for the proliferation of TITR mimics

**Fig. 7:**
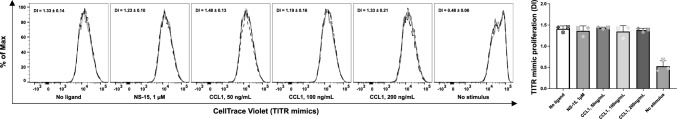
CCR8 is unnecessary for the proliferation of TITR mimics. Expanded Tregs were labeled with 2 µM CellTrace Violet and then cultured at 5 × 10^4^ cells per well in flat-bottom 96-well plates in TCM231-XF medium containing 12.5 μg/mL CD3/CD28 activator, 300 IU/mL IL-2 and 10 nM VitD3 for 4 days. CCR8 ligands at the indicated concentrations were added at 24 h, 48 h and 72 h. Left histograms show the cell proliferation in a single experiment with four technical replicates. Right bars show the division index (DI) calculated from three biological replicates (each performed in triplicate) by FlowJo’s proliferation modeling. Experiments were repeated three times. For bar graphs, a single independent experiment is represented by data points of the same shape (*n* = 3). Data shown as mean ± SD. No stimulus: cells were cultured in TCM231-XF medium without CD3/CD28 activator, IL-2 and VitD3. Histograms were generated as SVG files using FlowJo software. Bar graphs were created using GraphPad Prism. The alignment of the histograms and bar graphs was performed using the open-source Inkscape software

CCR8 has been implicated in the proliferation of Tregs [23], group 2 innate lymphoid cells [24] and Sézary cells [25]. It is of interest to investigate whether CCR8 ligands affect the proliferation of TITR mimics. Therefore, we labeled TITR mimics with CellTrace Violet and cultured them in TCM231-XF medium containing CD3/CD28 activator, IL-2 and VitD3 for 4 days. CCL1 or NS-15 were added to this culture at 24 h, 48 h and 72 h. The results showed that the proliferation of TITR mimics was not affected by addition of CCL1 and NS-15 (Fig. 7).

### CCR8 is not involved in the survival of TITR mimics

**Fig. 8:**
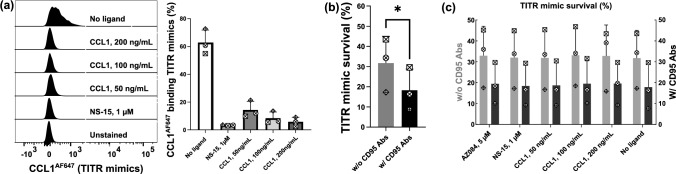
CCR8 is dispensable for the survival of TITR mimics. **a** CCL1^AF647^ binding on TITR mimics used for survival assay in the presence of CCR8 ligands. **b** The survival rate of TITR mimics in the presence or absence of CD95 antibody. **c** The survival rate of TITR mimics in the presence of CCR8 ligands. After 9 days of expansion, Tregs were grown in TCM231 containing 12.5 μg/mL CD3/CD28 activator, 300 IU/mL IL-2 and 10 nM VitD3 for another 3 days. At day 12, a partial of the obtained TITR mimics were stained with 3 nM CCL1^AF647^ after incubation with CCR8 ligands at the indicated concentrations for 30 min (**a**). The remaining cells were labeled with 1 μM CellTrace Violet for cell tracking. Afterward, the labeled cells treated with CCR8 ligands at the indicated concentration in the presence or absence of 2 μg/mL CD95 antibody for 24 h and then stained with APC Annexin V following the manufacturer’s protocol. The survival rate was calculated for CellTrace Violet-labeled cells. For bar graphs, a single experiment is represented by data points of the same shape (*n* = 3). Data shown as mean ± SD. **b** *p ≤ 0.05 by paired *t* test. Histograms were generated as SVG files using FlowJo software. Bar graphs were created using GraphPad Prism. The alignment of the histograms and bar graphs was performed using the open-source Inkscape software

It has been shown that CCR8 activation can rescue cancer cells from dexamethasone-induced apoptosis or potentiate the survival of Tregs in the presence of dendritic cells [26–28]. To investigate the role of CCR8 in the survival of TITR mimics, we cultured TITR mimics for 3 days in TCM231-XF medium containing CD3/CD28 activator, IL-2 and VitD3. Hereafter, some of the obtained cells were used to determine the expression of CCR8. The remaining cells were labeled with 1 µM CellTrace Violet and then cultured in XF medium with or without CD95 antibody in the presence of CCL1 or NS-15 for 24 h, where removing the stimulation factors and adding CD95 antibody would allow more apoptosis to occur. As shown in Fig. 8a, the expression of CCR8 was still observed in around 60% of Tregs. Analysis on CellTrace Violet-labeled cells showed that treatment with a CD95 antibody significantly decreased the survival rate of TITR mimics (Fig. 8b). However, neither CCL1 nor NS-15 had any effect on the survival of TITR mimics no matter the presence of CD95 antibody (Fig. 8c).

## Discussion

Despite significant advancements in the field of TITRs, a readily available and physiologically relevant cellular model for studying TITRs is still lacking. In the current study, we identified four factors, CD3/CD28 activator, IL-2, VitD3 and TCM, that facilitate the generation of highly immunosuppressive TITR mimics displaying high expression of TITR signature molecules (including CCR8) starting from healthy PBMC-derived Tregs. While CD3/CD28 activator, IL-2 and VitD3 have well-defined mechanisms of action, the functional factors within TCM that contribute to the TITR mimic phenotype and function remain undefined. Thus, it is of interest to determine whether TCM sourced from different cancer cell lines can be used to generate TITR mimics. To have a quick insight into this question, we performed a side-by-side comparison of TCM231 and TCM549 for their ability to upregulate TITR signature molecules. Our results revealed a comparable expression of a number of TITR signature molecules including CD30, CD39, CD134, CD137, TIGIT, Tim-3, CTLA-4, CD80, CD120b, CD122, GITR and ICOS (Fig. S5). Moreover, when PBMCs were co-cultured with Huh7 cells (hepatocellular carcinoma cells), the Treg population were also found to upregulate a series of TITR associated molecules [29]. These observations are consistent with previous findings that TITR signatures are conserved across different tumor types [17], suggesting the feasibility of generating TITR mimics through the utilization of various TCM formulations. Although beyond the scope of this study, the identification of the functional factors present in TCM would be of great interest in future as similar stimulatory components might also be present in vivo in the tumor microenvironment. One factor that might be worth looking into is Prostaglandin E2, considering its potential to be generated by cancer cells [30] and its documented ability to upregulate CCR8 in both naive and effector T cells [14, 15].

Previous studies showed that high doses of IL-2 can abrogate the immunosuppressive activity of Tregs [31–33]. Specifically, when exposed to IL-2 concentrations ranging from approximately 100 IU/mL–50 ng/mL (equivalent to around 1050 IU/mL), Tregs showed significantly diminished or undetectable immunosuppressive effect on Tresp proliferation. In contrast, our TITR mimics displayed robust immunosuppressive activity in the presence of 300 IU/mL of IL-2. A possible explanation for this discrepancy could be that the freshly isolated or short-term activated Tregs used in the aforementioned studies [31–33] may have low expression of CD25, as suggested by our Fig. S1, which hampers their ability to compete effectively with Tresps for IL-2 consumption. In contrast, our TITR mimics had much higher CD25 expression (Fig. S6), enabling them to outcompete Tresps in IL-2 consumption more effectively. With the heightened CD25 expression, it is highly probable that our TITR mimics also possess enhanced level of inhibitory TITR signature molecules by responding to the high concentration of IL-2, further enhancing their immunosuppressive activity compared to previously used Tregs.

Since chemokine receptors are typically involved in migration of immune cells between and within tissues, we examined the migratory ability of TITR mimics mediated by CCR8. A strong CCR8-specific response was observed, which suggests that TITR migration can be (co-)regulated by the highly induced CCR8 expression. However, it is important to note here that the TITRs also exhibit a high expression of CCR4 [1, 20–22], a chemokine receptor closely related to CCR8, which is also involved in controlling migration of Tregs in vivo [34]. Given that CCL1 and CCL22, chemokine ligands for CCR8 and CCR4, are both mainly secreted by myeloid cells in vivo [1, 35, 36] and blocking CCR8 evidently does not prevent CCR4-mediated migration toward CCL22 (Fig. 5c), it is likely that the effect of blocking CCR8 on TITR migration might (in part) be compensated for by CCR4. It has been shown that CCR8 is dispensable for Treg accumulation at tumor sites [7]. Despite this, it cannot be excluded that CCR8 may contribute to the coordinated migration and positioning of TITRs alongside CCR4 and other TITR-expressing chemokine receptors, which remains to be fully understood. It is also possible that CCR8 plays unique role in TITR migration toward yet-unidentified cells that primarily secrete CCL1 rather than other chemokines.

Our migration data also demonstrate that CCR8 expressed on the TITR mimics is a functional receptor that responds to its natural agonist CCL1 and thus induces CCR8 signaling in these cells. Our further study, however, indicated that this CCR8 signaling does not seem to be essential for the immunosuppressive activity of TITRs. This finding is in line with prior studies on the in vitro immunosuppressive capacity of TITRs. These studies have indicated that CCL1 stimulation does not affect the suppressive capacity of TITRs [1] and that TITRs derived from wild type and CCR8 knockout mice display similar suppressive capacities [3]. A possible explanation for this is that our TITR mimics, as well as TITRs, already possess strong immunosuppressive capacities (Fig. 6 and Fig. S4), which could be due to their abundant expression of immune checkpoint molecules, such as CD39, TIGIT, CTLA-4 and Tim3, as well as other possible functional mechanisms like inhibitory cytokines. Such strong immunosuppressive activity might be difficult to modulate by only targeting CCR8.

Moreover, our findings indicated that the CCR8 signaling also appears to be dispensable for Treg proliferation and apoptosis. This contradicts a study revealing a decrease in TITR number at tumor sites by IPG7236, a selective CCR8 antagonist [8]. However, it is in line with the majority of previous studies, which showed that CCR8-blocking antibodies did not impact TITR number [3–6]. The cited authors did not elucidate how IPG7236 reduces the TITR number. The substantial reduction in TITR numbers (around 80%, from roughly 2.7% to 0.5%) achieved by IPG7236 seems improbable to result solely from the inhibition of CCR8 signaling. Otherwise, it implies that CCR8 signaling plays a dominant role in TITR proliferation or survival, which contradicts prior studies using CCR8-blocking antibodies and our own data presented in Fig. 7. As our results showed, CD3/CD28 activation, IL-2 and potentially multiple other (yet to be identified) factors in TCM or the in vivo TME have already significantly facilitated TITR proliferation (Fig. 7). Given this, it is not unexpected that the impact of specifically modulating CCR8 signaling cannot be detected, if any.

The aim of our study was to generate a cellular model for TITRs that could be used to study the role of CCR8 signaling and would allow to evaluate the potential of small molecule CCR8 antagonists in future anti-tumor application. Even though antagonist NS-15 can efficiently block CCR8-mediated TITR mimic migration, its effect can be compensated by the function of CCR4. Moreover, NS-15 does not have any impact on the immunosuppressive activity, proliferation and survival of TITR mimics. Consequently, these findings raise doubts on the promise of CCR8 antagonists as potential anti-tumor agents. Considering the knowledge that the anti-tumor activity of CCR8 antibodies depends on their ADCC ability, a more promising future avenue for the development of CCR8-targeting small molecule candidates for cancer treatment may lie in the creation of small molecule drug conjugates (SMDCs) capable of selectively targeting and eliminating CCR8-expressing TITRs, which are prevalent in diverse tumor types.

Finally, apart from studying CCR8, we believe that our TITR mimics can be applied as a platform to identify and evaluate TITR-targeting drug candidates. The in vitro suppression assay holds promise for evaluating candidates targeting crucial immune checkpoint molecules involved in TITR function. Furthermore, the development of cell-killing assays also enables the assessment of ADCC-competent antibodies, antibody drug conjugates (ADCs) and SMDCs. Interestingly, our TITR mimics also allow the evaluation of drug candidate combinations, which we believe is a future direction for cancer treatment.

## Supplementary Information

Below is the link to the electronic supplementary material.

Supplementary file1 (DOCX 7987 KB)

## Data Availability

All data relevant to the study are included in the article or uploaded as supplementary information. All materials associated with this study are commercially available except for self-prepared Tregs, Tresps and small molecule NS-15. Data sharing is not applicable to this article as no datasets were generated or analyzed during the current study.

## Abbreviations

ADCC: Antibody-dependent cellular cytotoxicity
CCR8: CC chemokine receptor 8
ICI: Immune checkpoint inhibitor
IL-2: Interleukin-2
PBMC: Peripheral blood mononuclear cell
PD1: Programmed cell death protein 1
TCM: Tumor cell-conditioned medium
TCM-XF medium: Mixture of XF medium and TCM
TITR: Tumor-infiltrating regulatory T cells
VitD3: 1α,25-Dihydroxyvitamin D3
XF medium: ImmunoCult™-XF T cell expansion medium

## References

[1] Plitas G, Konopacki C, Wu K et al (2016) Regulatory T cells exhibit distinct features in human breast cancer. Immunity 45(5):1122–1134 27851913 10.1016/j.immuni.2016.10.032 PMC5134901

[2] De Simone M, Arrigoni A, Rossetti G et al (2016) Transcriptional landscape of human tissue lymphocytes unveils uniqueness of tumor-infiltrating T regulatory cells. Immunity 45(5):1135–1147 27851914 10.1016/j.immuni.2016.10.021 PMC5119953

[3] Van Damme H, Dombrecht B, Kiss M et al (2021) Therapeutic depletion of CCR8^+^ tumor-infiltrating regulatory T cells elicits antitumor immunity and synergizes with anti-PD-1 therapy. J Immunother Cancer 9(2):e001749 33589525 10.1136/jitc-2020-001749 PMC7887378

[4] Campbell JR, McDonald BR, Mesko PB et al (2021) Fc-optimized anti-CCR8 antibody depletes regulatory T cells in human tumor models. Cancer Res 81(11):2983–2994 33757978 10.1158/0008-5472.CAN-20-3585

[5] Kidani Y, Nogami W, Yasumizu Y et al (2022) CCR8-targeted specific depletion of clonally expanded Treg cells in tumor tissues evokes potent tumor immunity with long-lasting memory. Proc Natl Acad Sci USA 119(7):e2114282119 35140181 10.1073/pnas.2114282119 PMC8851483

[6] Weaver JD, Stack EC, Buggé JA et al (2022) Differential expression of CCR8 in tumors versus normal tissue allows specific depletion of tumor-infiltrating T regulatory cells by GS-1811, a novel Fc-optimized anti-CCR8 antibody. Oncoimmunology 11(1):2141007 36352891 10.1080/2162402X.2022.2141007 PMC9639568

[7] Whiteside SK, Grant FM, Gyori DS et al (2021) CCR8 marks highly suppressive Treg cells within tumours but is dispensable for their accumulation and suppressive function. Immunology 163(4):512–520 33838058 10.1111/imm.13337 PMC8274197

[8] Wu Y, Xi J, Li Y et al (2023) Discovery of a potent and selective CCR8 small molecular antagonist IPG7236 for the treatment of cancer. J Med Chem 66(7):4548–4564 36988587 10.1021/acs.jmedchem.3c00030

[9] Zhang Z, Wang G, Shao X et al (2023) A novel prognostic biomarker CCR8 for gastric cancer and anti-CCR8 blockade attenuate the immunosuppressive capacity of Tregs *In Vitro*. Cancer Biother Radiopharm. 10.1089/cbr.2022.0095 37102694 10.1089/cbr.2022.0095

[10] Liu L, Doijen J, D’huys T et al (2021) Biological characterization of ligands targeting the human CC chemokine receptor 8 (CCR8) reveals the biased signaling properties of small molecule agonists. Biochem Pharmacol 188:114565 33872569 10.1016/j.bcp.2021.114565

[11] Putnam AL, Brusko TM, Lee MR et al (2009) Expansion of human regulatory T-cells from patients with type 1 diabetes. Diabetes 58(3):652–662 19074986 10.2337/db08-1168 PMC2646064

[12] Revenko A, Carnevalli LS, Sinclair C et al (2022) Direct targeting of FOXP3 in Tregs with AZD8701, a novel antisense oligonucleotide to relieve immunosuppression in cancer. J Immunother Cancer 10(4):e003892 35387780 10.1136/jitc-2021-003892 PMC8987763

[13] Zorn E, Nelson EA, Mohseni M et al (2006) IL-2 regulates FOXP3 expression in human CD4^+^CD25^+^ regulatory T cells through a STAT-dependent mechanism and induces the expansion of these cells in vivo. Blood 108(5):1571–1579 16645171 10.1182/blood-2006-02-004747 PMC1895505

[14] McCully ML, Collins PJ, Hughes TR et al (2015) Skin metabolites define a new paradigm in the localization of skin tropic memory T Cells. J Immunol 195(1):96–104 26002980 10.4049/jimmunol.1402961 PMC4472944

[15] Fraga M, Yáñez M, Sherman M et al (2021) Immunomodulation of T helper cells by tumor microenvironment in oral cancer is associated with CCR8 expression and rapid membrane vitamin D signaling pathway. Front Immunol. 10.3389/fimmu.2021.643298 34025655 10.3389/fimmu.2021.643298 PMC8137990

[16] Mijnheer G, Lutter L, Mokry M et al (2021) Conserved human effector Treg cell transcriptomic and epigenetic signature in arthritic joint inflammation. Nat Commun 12(1):2710 33976194 10.1038/s41467-021-22975-7 PMC8113485

[17] Magnuson AM, Kiner E, Ergun A et al (2018) Identification and validation of a tumor-infiltrating Treg transcriptional signature conserved across species and tumor types. Proc Natl Acad Sci 115(45):E10672–E10681 30348759 10.1073/pnas.1810580115 PMC6233093

[18] Miller MD, Hata S, De Waal MR et al (1989) A novel polypeptide secreted by activated human T lymphocytes. J Immunol (Baltim Md 1950) 143(9):2907–2916 2809212

[19] Jenkins TJ, Guan B, Dai M et al (2007) Design, synthesis, and evaluation of naphthalene-sulfonamide antagonists of human CCR8. J Med Chem 50(3):566–584 17266208 10.1021/jm061118e

[20] Sarkar T, Dhar S, Chakraborty D et al (2022) FOXP3/HAT1 axis controls treg infiltration in the tumor microenvironment by inducing CCR4 expression in breast cancer. Front Immunol. 10.3389/fimmu.2022.740588 35222362 10.3389/fimmu.2022.740588 PMC8863663

[21] Wang L, Simons DL, Lu X et al (2019) Connecting blood and intratumoral Treg cell activity in predicting future relapse in breast cancer. Nat Immunol 20(9):1220–1230 31285626 10.1038/s41590-019-0429-7 PMC8802768

[22] Sugiyama D, Nishikawa H, Maeda Y et al (2013) Anti-CCR4 mAb selectively depletes effector-type FoxP3^+^CD4^+^ regulatory T cells, evoking antitumor immune responses in humans. Proc Natl Acad Sci 110(44):17945–17950 24127572 10.1073/pnas.1316796110 PMC3816454

[23] Barsheshet Y, Wildbaum G, Levy E et al (2017) CCR8^+^FOXp3^+^ Treg cells as master drivers of immune regulation. Proc Natl Acad Sci 114(23):6086–6091 28533380 10.1073/pnas.1621280114 PMC5468670

[24] Knipfer L, Schulz-Kuhnt A, Kindermann M et al (2019) A CCL1/CCR8-dependent feed-forward mechanism drives ILC2 functions in type 2–mediated inflammation. J Exp Med 216(12):2763–2777 31537642 10.1084/jem.20182111 PMC6888976

[25] Giustiniani J, Dobos G, Moins-Teisserenc H et al (2022) CCR8 is a new therapeutic target in cutaneous T-cell lymphomas. Blood Adv 6(11):3507–3512 35201316 10.1182/bloodadvances.2021006512 PMC9198911

[26] Louahed J, Struyf S, Demoulin JB et al (2003) CCR8-dependent activation of the RAS/MAPK pathway mediates anti-apoptotic activity of I-309/ CCL1 and vMIP-I. Eur J Immunol 33(2):494–501 12645948 10.1002/immu.200310025

[27] Coghill JM, Fowler KA, West ML et al (2013) CC chemokine receptor 8 potentiates donor Treg survival and is critical for the prevention of murine graft-versus-host disease. Blood 122(5):825–836 23798714 10.1182/blood-2012-06-435735 PMC3731935

[28] Spinetti G, Bernardini G, Camarda G et al (2003) The chemokine receptor CCR8 mediates rescue from dexamethasone-induced apoptosis via an ERK-dependent pathway. J Leukoc Biol 73(1):201–207 12525579 10.1189/jlb.0302105

[29] Cao M, Cabrera R, Xu Y et al (2007) Hepatocellular carcinoma cell supernatants increase expansion and function of CD4^+^CD25^+^ regulatory T cells. Lab Invest 87(6):582–590 17372588 10.1038/labinvest.3700540

[30] Finetti F, Travelli C, Ercoli J et al (2020) Prostaglandin E2 and cancer: insight into tumor progression and immunity. Biology 9(12):434 33271839 10.3390/biology9120434 PMC7760298

[31] Takahashi T, Kuniyasu Y, Toda M et al (1998) Immunologic self-tolerance maintained by CD25^+^CD4^+^ naturally anergic and suppressive T cells: induction of autoimmune disease by breaking their anergic/suppressive state. Int Immunol 10(12):1969–1980 9885918 10.1093/intimm/10.12.1969

[32] de la Rosa M, Rutz S, Dorninger H et al (2004) Interleukin-2 is essential for CD4^+^CD25^+^ regulatory T cell function. Eur J Immunol 34(9):2480–2488 15307180 10.1002/eji.200425274

[33] Moon BI, Kim TH, Seoh JY (2015) Functional modulation of regulatory T cells by IL-2. PLoS One 10(11):e0141864 26529512 10.1371/journal.pone.0141864 PMC4631326

[34] Kohli K, Pillarisetty VG, Kim TS (2022) Key chemokines direct migration of immune cells in solid tumors. Cancer Gene Ther 29(1):10–21 33603130 10.1038/s41417-021-00303-x PMC8761573

[35] Anz D, Rapp M, Eiber S et al (2015) Suppression of intratumoral CCL22 by type I interferon inhibits migration of regulatory T cells and blocks cancer progression. Cancer Res 75(21):4483–4493 26432403 10.1158/0008-5472.CAN-14-3499

[36] Wiedemann GM, Knott MML, Vetter VK, et al. (2016) Cancer cell-derived IL-1α induces CCL22 and the recruitment of regulatory T cells. Oncoimmunology [Internet]. [cited 2023 Oct 20];5(9). Available from: https://www.ncbi.nlm.nih.gov/pmc/articles/PMC5048775/10.1080/2162402X.2016.1175794 PMC5048775 27757295

