# Supplementary material for: Development of a cellular model to study CCR8 signaling in tumor-infiltrating regulatory T cells
Source: Cancer Immunol Immunother. 2024 Jan 17;73(1):11. doi: 10.1007/s00262-023-03607-z (PMC10794316; doi:10.1007/s00262-023-03607-z)
Supplement: Supplementary file 1 — Supplementary file1 (DOCX 7987 KB) [file 262_2023_3607_MOESM1_ESM.docx]

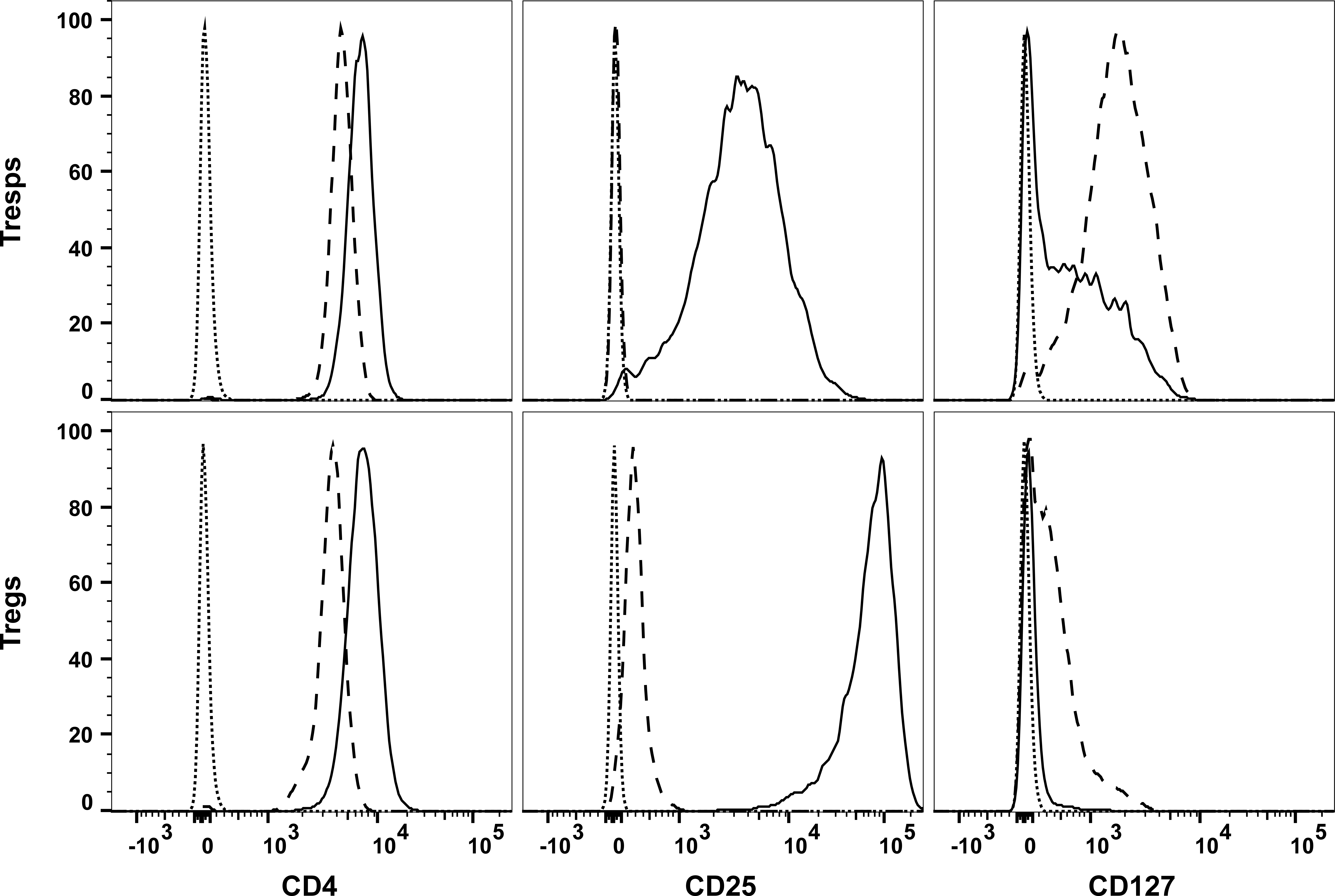


**Fig. S1** Characterization of freshly and expanded Tresps and Tregs. Tregs and Tresps were stained with PerCP-CD4 at 1:100 (Biolegend, #344624), PE-CD25 at 1:20 (BD, #555432), AF647-CD127 at 1:10 (BD, #558598) immediately after isolation (dashed) or after expansion for 7 days (solid). Unstained cells were used as negative control (dottted). Fresh Tresps did not express CD25 but had a high level of CD127 expression, whereas fresh Tregs were CD25 positive and expressed CD127 at low level. After expansion, both Tregs and Tresps experienced a significant increase in CD25 expression and decrease in the expression of CD127.





**Fig. S2** Representative histograms showing the expression of several TITR signature molecules in Tregs. After expansion for 9 days, Tregs were grown in XF medium or TCM231-XF medium containing different combination of 12.5 μg/mL CD3/CD28 activator, 300 IU/mL IL-2 and 10 nM VitD3 for 3 days. The obtained cells were then stained with corresponding antibodies followed by flow cytometry analysis.


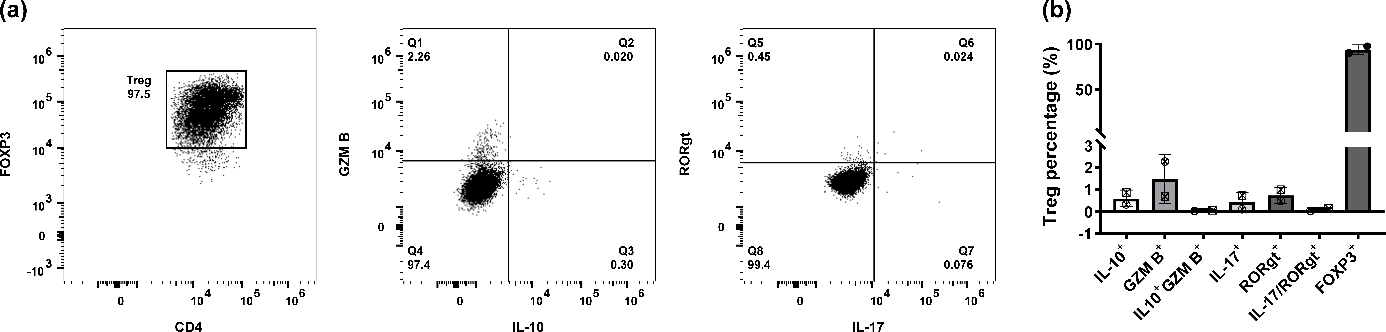


**Fig. S3** The expression analysis of FOXP3 and cytokines in TITR mimics. (a) Gating strategy for evaluation of FOXP3 and cytokines. (b) The percentage of TITRs mimics expressing FOXP3 and cytokines (n=2). Tregs that underwent 9 days of expansion were restimulated with 12.5 µL/mL CD3/CD28 activator, 300 IU/mL IL-2 and 10 nM VitD3 in TCM231-XF medium. Four days later, the cells were stimulated for 4 hours at 37°C with 50ng/ml Phorbol 12,13-dibutyrate (Tocris, #4153 ), 1µg/ml Ionomycin (Tocris, #1704) and 1µg/ml Brefeldin-A (Tocris, #1231). The cells were treated with FcR-Block (BD, #564220) and live dead blue staining (Thermofisher, #L23105) for 20min at 4°C. Then fixation and permeabilization was done using the FOXP3 Transcription Factor Staining Buffer Set (Thermofisher, #00522356) according to the manufacturer’s instructions. Afterwards, the cells were stained at 4°C overnight with antibodies for CD3 (Biolegend, #300425), CD4 (Invitrogen, #48004842), CD8 (Biolegend, #344724), FOXP3 (Sony, #2200560), IL-10 (Sony, #3134055), granzyme B (Biolegend, #515406), RORgt (BD, #563081) and IL-17 (Sony, #3161615). The samples were acquired on the SonyID7000 and Cytek Aurora.CD3^+^CD4^+^CD8^-^ cells were analyzed on Flowjo (version 10.9.0).

**Fig. S4** TITR mimics display enhanced suppressive capability. (a) Representative histograms of two independent experiments showing proliferation profiles of Tresps in the presence of different doses of TITR mimics (in TCM231-XF medium) or Tregs (in XF medium), along with 12.5 µL/mL CD3/CD28 activator, 300 IU/mL IL-2 and 10 nM VitD3 for 4 days. Tregs were expanded for 9 days before use as described in the main text. Tresps were also expanded for 9 days but were stimulated with 10µL/mL CD3/CD28 activator and 210 IU/mL at day 0. The volume was increased by threefold at day 2, then fourfold at day 5 and day 7. IL-2 was always supplemented at a final concentration of 210 IU/mL at day2/5/7. Unstimulated Tresps were used as control. (b) The division index of Tresps from the two independent experiments. A single independent experiment is represented by the data point with same shape. (c) The inhibition percentage of Tresp proliferation by TITR mimics or Tregs. The calculation of inhibition percentage follows the formula: (DI_0:1_-DI_1:x_)/DI_0:1_*100, in which DI_0:1_ means the division index of Tresps in the absence of TITR mimics or Tregs, DI_1:x_ means the division index of Tresps in the presence different doses of TITR mimics or Tregs.


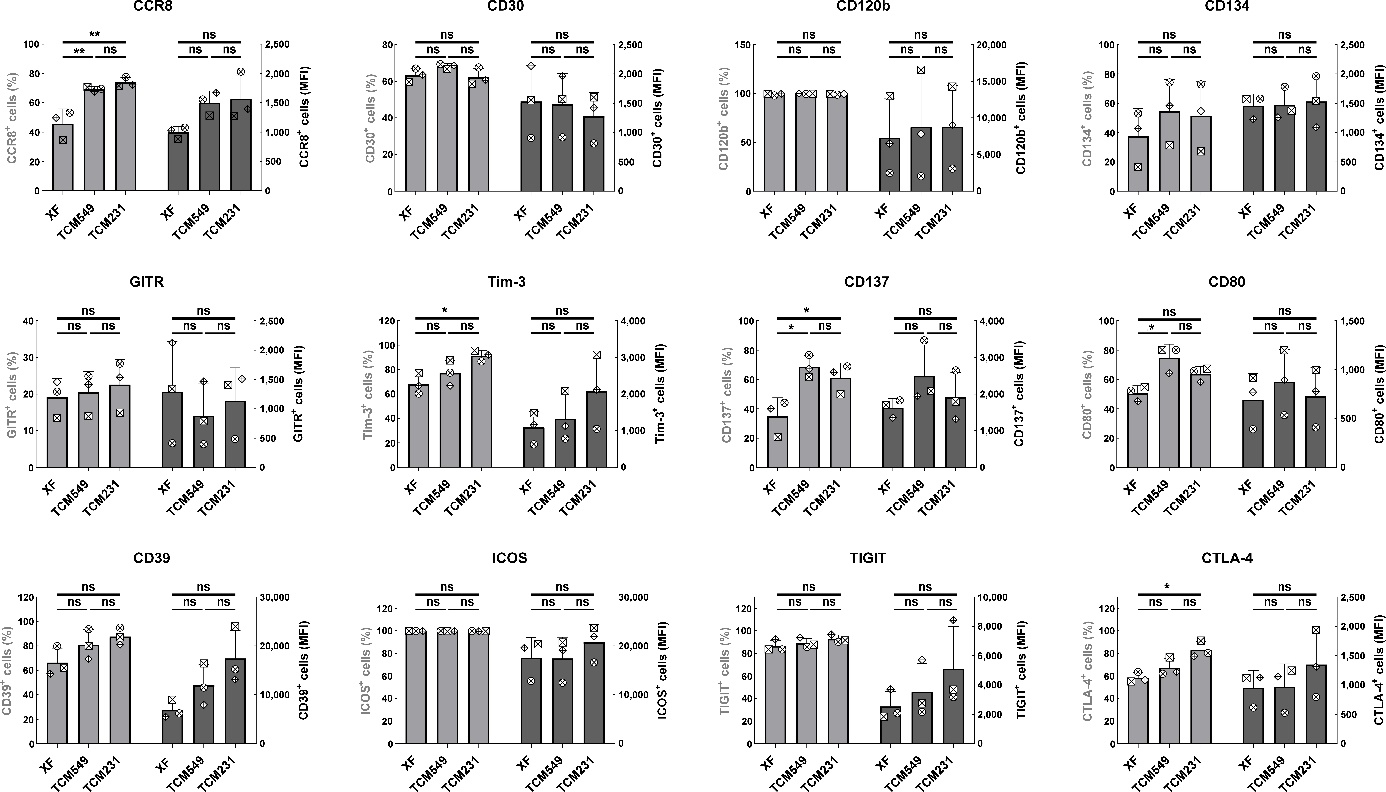


**Fig. S5** TITR signature molecules were conserved in TITR mimics generated using TCM derived from different cancer cells. After 9 days of expansion, Tregs were grown in the presence of 12.5 µL/mL CD3/CD28 activator plus 300 IU/mL IL-2 for 3 days in XF, TCM231-XF or TCM549-XF medium. The cells were then stained with antibodies targeting indicated TITR signature molecules followed by analysis using flow cytometry. The percentage and median fluorescence intensity of the cells expressing these molecules were shown (n=3). A single experiment is represented by the data point with same shape. * p ≤ 0.05, ** p ≤ 0.01 by paired t test.


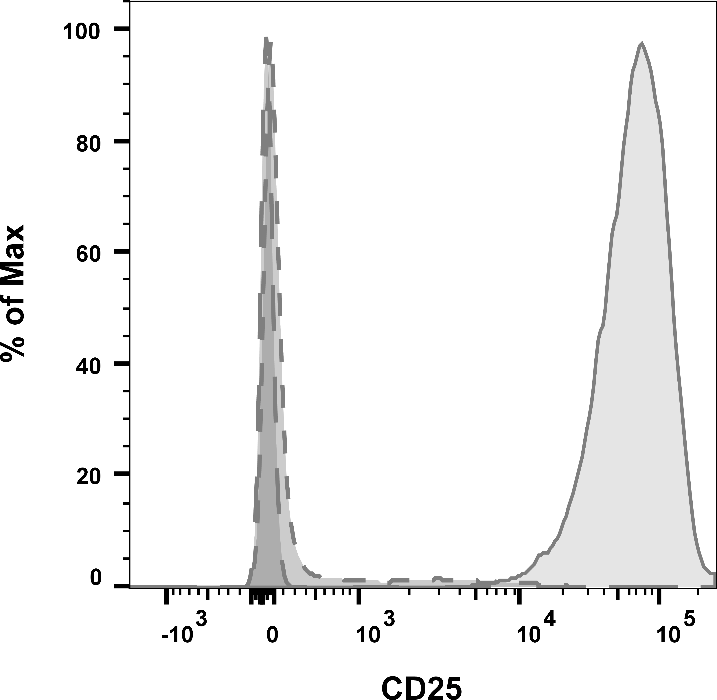


**Fig. S6** CD25 expression in TITR mimics. After nine days of expansion, Tregs were restimulated for 24 hours in TCM231-XF medium containing 12.5 µL/mL CD3/CD28 activator, 300 IU/mL IL-2 and 10 nM VitD3. Then the cells were stained with CD25 antibody (solid line). Isotype staining (dashed line) and unstained (long-dash line) were showed as control.
